# Factors Associated With Long‐Term Visual Field Variability in Patients With Normal‐Tension Glaucoma

**DOI:** 10.1155/joph/7211382

**Published:** 2026-03-23

**Authors:** Seunghee Ha, Sangwoo Moon, EunAh Kim, Sanghun Jeong, Hojin Yang, Jiwoong Lee

**Affiliations:** ^1^ Department of Ophthalmology, Pusan National University School of Medicine, Busan, South Korea, pusan.ac.kr; ^2^ Department of Ophthalmology, Pusan National University Yangsan Hospital, Pusan National University School of Medicine and Research Institute for Convergence of Biomedical Science and Technology, Yangsan, South Korea, pnuyh.or.kr; ^3^ Department of Ophthalmology, Samsung Changwon Hospital, Sungkyunkwan University School of Medicine, Changwon, South Korea, skku.edu; ^4^ Department of Statistics, Changwon National University, Changwon, South Korea, changwon.ac.kr; ^5^ Department of Statistics, Pusan National University, Busan, South Korea, pusan.ac.kr; ^6^ Biomedical Research Institute, Pusan National University Hospital, Busan, South Korea, pnuh.or.kr

**Keywords:** intraocular pressure, normal-tension glaucoma, optical coherence tomography, retinal nerve fiber layer thickness, visual field test

## Abstract

**Purpose:**

To investigate the factors associated with variability in the longitudinal visual field (VF) test in patients with normal‐tension glaucoma (NTG).

**Methods:**

This retrospective study enrolled patients with NTG who underwent ≥ 12 reliable VF tests over a follow‐up period of ≥ 6 years. For each eye, 52 total deviation values (TDVs) were linearly regressed against time (years), while the root‐mean‐squared error (RMSE) of the residuals was calculated to quantify the long‐term VF variability. Hierarchical cluster analysis using squared Spearman correlation coefficients was conducted to assess collinearity and select variables. Six linear mixed‐effects models were constructed, each including clinical parameters such as intraocular pressure (IOP), retinal nerve fiber layer thickness (RNFLT), and VF parameters. Mixed‐effects regression was applied to evaluate the factors associated with long‐term VF variability, adjusting for intereye correlation.

**Results:**

The study analyzed 114 eyes of 77 patients with NTG. In models including baseline RNFLT, lower baseline IOP, lower mean IOP, greater IOP fluctuation, thinner baseline RNFLT, and a steeper mean deviation (MD) slope were significantly associated with increased long‐term VF variability (all *p* ≤ 0.031). In models that included baseline MD, worse baseline MD and a steeper MD slope were significantly associated with increased long‐term VF variability (all *p* ≤ 0.004).

**Conclusions:**

Lower baseline and mean IOP, greater IOP fluctuation, thinner baseline RNFLT, worse baseline MD, and steeper MD slope are all associated with a greater long‐term VF variability in patients with NTG. NTG eyes with thin RNFL or rapid VF deterioration may require more frequent VF testing to avoid misinterpreting fluctuation as true progression.

## 1. Introduction

Normal‐tension glaucoma (NTG) is a form of glaucomatous optic neuropathy with corresponding visual field (VF) defects occurring at untreated intraocular pressure (IOP) of 21 mmHg or less. NTG is the most common form of open‐angle glaucoma in East Asian populations [1]. Because NTG progresses without an elevated IOP, structural and functional monitoring, particularly through VF testing, plays a critical role in disease management. The early detection of VF progression and appropriate treatment initiation are essential to prevent irreversible vision loss in these patients [2, 3].

Given its clinical importance, standard automated perimetry is widely applied as the gold standard for the assessment of glaucomatous progression [4]. However, VF test results can be influenced by both intrinsic and extrinsic factors, leading to long‐term variability in the standard automated perimetry outcomes. This variability is known to be associated with reliability indices, including false‐positive and false‐negative rates, as well as patient‐related factors including fatigue, learning effects, and media opacity [5–8]. Such fluctuations may alternately obscure true disease progression or result in the overestimation of disease worsening, thereby complicating clinical decision‐making [5, 6].

In a prior study, Rabiolo et al. analyzed the factors influencing long‐term VF variability in patients with glaucoma and reported that greater IOP fluctuations, worse baseline mean deviation (MD), higher false‐positive and false‐negative rates, history of glaucoma surgery during follow‐up, faster VF deterioration, longer follow‐up duration, and more frequent testing were all associated with increased variability [6]. However, that study did not incorporate structural parameters such as optical coherence tomography (OCT) findings. Moreover, there is a lack of research specifically investigating long‐term VF variability in patients with NTG in clinical settings [9]. The present study specifically focused on investigating patients with NTG to address the limitations of previous studies.

This study aimed to investigate the clinical and structural factors associated with long‐term variability of standard automated perimetry in patients with NTG.

## 2. Methods

### 2.1. Study Population

This retrospective study analyzed the medical records of patients diagnosed with NTG who had been followed up for more than six years and had undergone at least 12 VF examinations at our institution. This study was approved by the Institutional Review Board of the Pusan National University Hospital (approval number: 2309‐029‐131) and was conducted in adherence to the tenets of the Declaration of Helsinki [10].

At the baseline visit, all patients underwent comprehensive ophthalmic evaluation, including slit‐lamp biomicroscopy, gonioscopy, IOP measurement using Goldmann applanation tonometry, best‐corrected visual acuity (BCVA) assessment, and ocular biometry using the IOL Master (Carl Zeiss Meditec, Dublin, CA, USA) to determine central corneal thickness and axial length. VF testing was conducted using a Humphrey Field Analyzer (Carl Zeiss Meditec) with the 24‐2 SITA Standard strategy. After adequate pupillary dilation was achieved, spectral‐domain OCT imaging was performed using Cirrus OCT (software version 4.0; Carl Zeiss Meditec) with the Optic Disc Cube protocol. This protocol acquired a cube of data over a 6 × 6 mm square area centered on the optic disc. A total of 200 horizontal B‐scans were obtained, with each B‐scan composed of 200 A‐scans, resulting in a dense three‐dimensional data set of the optic nerve head and peripapillary region. From these data, the peripapillary retinal nerve fiber layer thickness (RNFLT) was automatically calculated along a 3.46‐mm diameter calculation circle centered on the optic disc. Only scans with adequate quality (signal strength ≥ 6, proper centration, and free of motion or segmentation artifacts) were included.

The baseline IOP was defined as the IOP measured on the same day as the first eligible VF test. For patients who underwent intraocular surgery throughout the follow‐up period, IOP values and VF tests performed within three months of surgery were excluded to minimize any postoperative fluctuations. Postoperative IOP values were collected at least 3 months after surgery.

The mean IOP, IOP fluctuation (defined as the standard deviation of IOP measured across all visits), and peak IOP (defined as the highest IOP value recorded during the follow‐up period) were calculated using the IOP values measured with the Goldmann applanation tonometry.

To reduce learning effect, the first two VF tests were excluded. Only reliable VFs, defined as those with fixation losses ≤ 30%, false‐negative errors ≤ 30%, and false‐positive errors ≤ 15%, were included.

This study included only patients with naïve NTG, defined by an untreated IOP of ≤ 21 mmHg at baseline, with intervals of 2 h from 9 a.m. to 5 p.m., along with glaucomatous optic nerve damage or RNFL defects accompanied by corresponding VF abnormalities.

Glaucomatous optic nerve damage was defined by the presence of neuroretinal rim thinning, notching, optic disc hemorrhage, an intereye vertical cup‐to‐disc ratio difference of ≥ 0.2, or the presence of RNFL defects. Glaucomatous VF defects were defined as three or more contiguous points on the pattern deviation plot with sensitivity below the 5% probability level, at least one of which was below the 1% probability level, and an abnormal glaucoma hemifield test (outside normal limits) or pattern standard deviation (PSD) with a significance level below 5%.

The exclusion criteria were age ≤ 18 years, history of ocular trauma or intraocular surgery except for uncomplicated cataract extraction surgery, retinal or neurological diseases affecting vision or VFs, media opacities, and other optic neuropathies unrelated to glaucoma. When both eyes met the inclusion criteria, both were analyzed, and the intereye correlation was adjusted using linear mixed models.

### 2.2. Long‐Term VF Variability

To evaluate the long‐term VF variability, the root‐mean‐squared errors (RMSEs) of the total deviation values (TDVs) were calculated across all 52 test points, excluding the two blind spot locations, throughout the follow‐up period. For each eye, a linear regression was performed at each of the 52 test locations to model the change in TDV over time, from the first to the last VF test, using the following model:

*y* = *α* + *β* *x* where *x* is time (years) and *y* is the TDV (decibels).

The RMSE was then calculated as follows:

(1) RMSEi=∑t=1ntrueTDVi,t−predictedTDVi,t2n,

where RMSE_*i*_ is the root‐mean‐square error for the *i* th test location of VF exam, true*T* *D* *V*_*i*,*t*_ is the observed TDV at time *t*, predicted*T* *D* *V*_*i*,*t*_ is the predicted TDV from the regression model, and *n* is the number of VF examinations.

The mean of RMSE values from all 52 locations was then calculated to represent the long‐term VF variability for each eye.

### 2.3. Statistical Analysis

**FIGURE 1:**
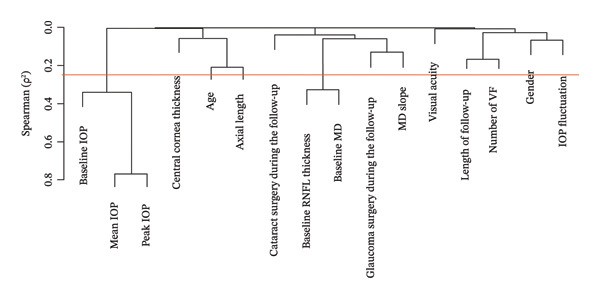
Results of hierarchical cluster analysis based on squared Spearman correlation coefficients used to assess collinearity between candidate variables prior to model fitting. The red line indicates the collinearity threshold of *ρ* ^2^ = 0.25. Abbreviations: IOP (intraocular pressure), MD (mean deviation), RNFL (retinal nerve fiber layer), VF (visual field).

All statistical analyses were conducted using the R software (R Foundation for Statistical Computing, Vienna, Austria; Version 4.1.1) [11]. To explore the potential collinearity among candidate variables, hierarchical cluster analysis was conducted using squared Spearman correlation coefficients (*ρ* ^2^) as a similarity metric. Redundancy was assessed using a threshold of *ρ* ^2^ ≤ 0.25, while variables considered clinically less relevant were excluded from further analysis (Figure 1).

The following baseline variables were included in all of the models: age, sex, BCVA, central corneal thickness, and axial length. The longitudinal variables that were common included the length of follow‐up, number of VF examinations, IOP fluctuation, whether the patient underwent cataract or glaucoma surgery during the follow‐up period, and MD slope.

In addition, the baseline RNFLT or baseline VF MD was included in each model. For IOP, one or more of the following variables were included in various combinations: baseline IOP, mean IOP, and peak IOP measured during follow‐up. Based on these combinations, six linear mixed models were constructed.

In all models, the RMSE of the TDV was set as the dependent variable. To account for the intereye correlation in patients with contributions to both eyes, a linear mixed model was fitted to the data, including a random effect on the subject. Statistical significance was set at *p* < 0.05.

The linear mixed model was adopted in this study for several reasons. First, it effectively accommodates correlated observations that arise from repeated measurements or clustered data structures, providing an appropriate framework for modeling within‐subject dependencies. Second, by incorporating random effects, it flexibly captures subject‐specific deviations from the population‐level trends, allowing individual heterogeneity to be explicitly modeled. Third, the model can handle unbalanced or missing data without discarding incomplete cases, thereby preserving statistical power. Moreover, it enables the specification of diverse covariance structures, which enhances the accuracy of variance component estimation and improves model fit compared to conventional linear models.

## 3. Results

A total of 114 eyes from 77 patients diagnosed with NTG who visited the Glaucoma Clinic at Pusan National University Hospital between June 2007 and January 2021 were included in the analysis. The mean (± standard deviation) age of the patients was 58.96 ± 15.85 years, and the average follow‐up period was 7.98 ± 1.35 years. Each eye underwent an average of 12.65 ± 2.03 VF examinations. The mean baseline VF MD was −4.49 ± 4.10 dB. The average baseline RNFLT was 79.27 ± 13.56 μm. The baseline, peak, and fluctuation in the IOP were 14.93 ± 2.83 mmHg, 18.74 ± 3.15 mmHg, and 1.91 ± 0.69 mmHg, respectively (Table 1).

**TABLE 1:** Demographic and clinical characteristics of the study population.

Characteristic	Value
Number of eyes	114
Number of patients	77
Age (years, mean ± SD)	58.96 ± 15.85
Gender (male:female)	55:59
Visual acuity (logMAR, mean ± SD)	0.17 ± 0.20
Central cornea thickness (μm, mean ± SD)	553.3 ± 33.03
Axial length (mm, mean ± SD)	24.84 ± 1.69
Follow‐up (years, mean ± SD)	7.98 ± 1.35
Number of visual field (mean ± SD)	12.65 ± 2.03
Baseline mean deviation (decibel, mean ± SD)	−4.49 ± 4.10
Baseline IOP (mmHg, mean ± SD)	14.93 ± 2.83
Mean IOP (mmHg, mean ± SD)	15.45 ± 2.25
IOP fluctuation (mmHg, mean ± SD)	1.91 ± 0.69
Peak IOP (mmHg, mean ± SD)	18.74 ± 3.15
Baseline retinal nerve fiber layer thickness (mean ± SD)	79.27 ± 13.56
Cataract surgery during the follow‐up (*n*, %)	13 (8.9)
Glaucoma surgery during the follow‐up (*n*, %)	13 (8.9)
Mean deviation slope (dB/year, mean ± SD)	−0.06 ± 0.32

*Note:* logMAR (logarithm of the minimum angle of resolution), IOP (intraocular pressure).

Abbreviation: SD, standard deviation.

**FIGURE 2:**
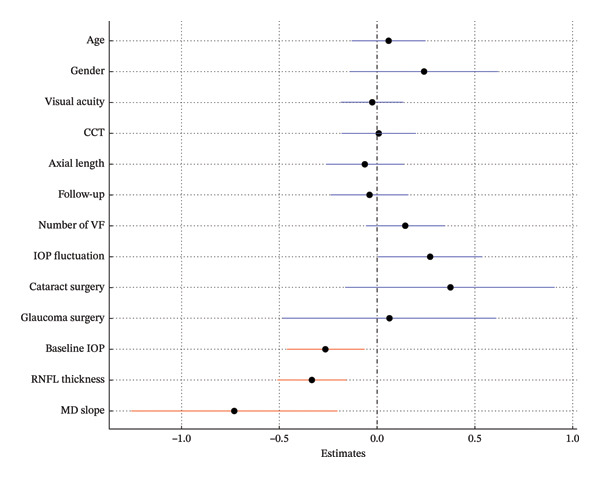
Forest plot showing Model 1, which includes baseline retinal nerve fiber layer (RNFL) thickness and baseline intraocular pressure (IOP). Dots indicate the estimates for the RMSE of the total deviation value (TDV); bars represent 95% confidence intervals. A lower baseline IOP, thinner baseline RNFL, and steeper MD slope were significantly associated with increased VF variability. Red markers indicate statistically significant variables (*p* < 0.05). Abbreviations: CCT, central corneal thickness; IOP, intraocular pressure; RNFL, retinal nerve fiber layer; VF, visual field.

**FIGURE 3:**
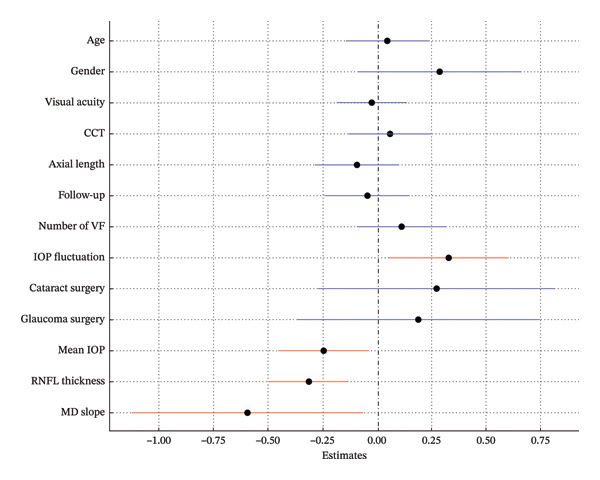
Forest plot showing Model 2, which includes baseline retinal nerve fiber layer (RNFL) thickness and mean intraocular pressure (IOP). Dots indicate the estimates for the RMSE of the total deviation value (TDV); bars represent 95% confidence intervals. A thinner baseline RNFL, steeper MD slope, lower mean IOP, and greater IOP fluctuation were significantly associated with increased VF variability. Red markers indicate statistically significant variables (*p* < 0.05). Abbreviations: CCT (central corneal thickness), IOP (intraocular pressure), RNFL (retinal nerve fiber layer), VF (visual field).

**FIGURE 4:**
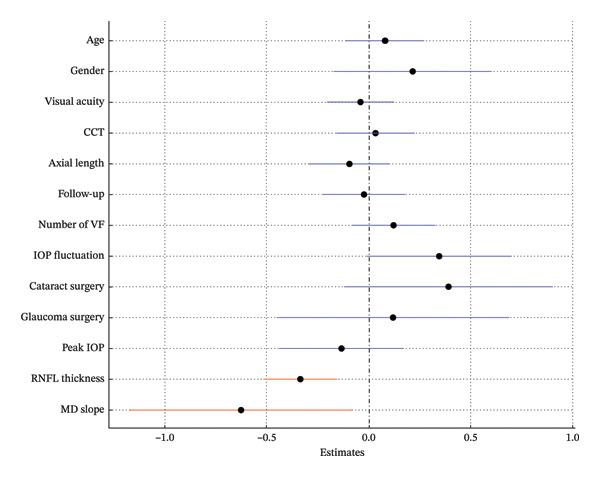
Forest plot showing Model 3, which includes baseline retinal nerve fiber layer (RNFL) thickness and peak intraocular pressure (IOP). Dots indicate the estimates for the RMSE of the total deviation value (TDV); bars represent 95% confidence intervals. A thinner baseline RNFL and steeper MD slope were significantly associated with increased VF variability. Red markers indicate statistically significant variables (*p* < 0.05). Abbreviations: CCT (central corneal thickness), IOP (intraocular pressure), RNFL (retinal nerve fiber layer), VF (visual field).

In Model 1, which included baseline RNFLT and IOP, lower baseline IOP, thinner baseline RNFLT, and a steeper MD slope were associated with a greater long‐term VF variability (*p* = 0.009, *p* < 0.001, *p* = 0.008; Table 2; Figure 2). In Model 2, which included the baseline RNFLT and mean IOP, a thinner baseline RNFLT, steeper MD slope, lower mean IOP, and greater IOP fluctuation were associated with increased long‐term VF variability (*p* < 0.001, *p* = 0.031, *p* = 0.022, *p* = 0.021; Table 3; Figure 3). In Model 3, which included baseline RNFLT and peak IOP, a thinner baseline RNFLT and a steeper MD slope were associated with increased long‐term VF variability (*p* < 0.001 and *p* = 0.027, respectively; Table 4; Figure 4).

**TABLE 2:** Relationship between long‐term pointwise variability and predictive factors of Model 1 (including baseline retinal nerve fiber layer thickness and baseline intraocular pressure).

	Estimate	Standard error	*p* value^∗^
Age	0.060	0.097	0.538
Gender	0.240	0.193	0.219
Visual acuity	−0.025	0.081	0.763
Central cornea thickness	0.008	0.097	0.931
Axial length	−0.063	0.101	0.532
Length of follow‐up	−0.038	0.101	0.710
Number of visual field	0.144	0.102	0.163
Intraocular pressure fluctuation	0.271	0.136	0.050
Cataract surgery during the follow‐up	0.374	0.274	0.176
Glaucoma surgery during the follow‐up	0.063	0.278	0.820
Baseline intraocular pressure	−0.265	0.099	0.009
Baseline retinal nerve fiber layer thickness	−0.333	0.090	< 0.001
Mean deviation slope	−0.731	0.270	0.008

^∗^ *p* value by linear mixed model.

**TABLE 3:** Relationship between long‐term pointwise variability and predictive factors of Model 2 (including baseline retinal nerve fiber layer thickness and mean intraocular pressure).

	Estimate	Standard error	*p* value^∗^
Age	0.048	0.097	0.619
Gender	0.287	0.193	0.143
Visual acuity	−0.023	0.082	0.776
Central cornea thickness	0.058	0.096	0.551
Axial length	−0.091	0.098	0.358
Length of follow‐up	−0.043	0.100	0.665
Number of visual field	0.111	0.101	0.275
Intraocular pressure fluctuation	0.329	0.141	0.021
Cataract surgery during the follow‐up	0.272	0.278	0.330
Glaucoma surgery during the follow‐up	0.189	0.284	0.506
Mean intraocular pressure	−0.245	0.105	0.022
Baseline retinal nerve fiber layer thickness	−0.311	0.091	0.001
Mean deviation slope	−0.595	0.272	0.031

^∗^ *p* value by linear mixed model.

**TABLE 4:** Relationship between long‐term pointwise variability and predictive factors of Model 3 (including baseline retinal nerve fiber layer thickness and peak intraocular pressure).

	Estimate	Standard error	*p* value^∗^
Age	0.082	0.098	0.410
Gender	0.217	0.198	0.278
Visual acuity	−0.041	0.084	0.63
Central cornea thickness	0.035	0.099	0.727
Axial length	−0.093	0.102	0.366
Length of follow‐up	−0.022	0.103	0.831
Number of visual field	0.123	0.105	0.244
Intraocular pressure fluctuation	0.347	0.181	0.058
Cataract surgery during the follow‐up	0.329	0.294	0.266
Glaucoma surgery during the follow‐up	0.121	0.291	0.678
Peak intraocular pressure	−0.132	0.156	0.399
Baseline retinal nerve fiber layer thickness	−0.334	0.093	0.001
Mean deviation slope	−0.626	0.278	0.027

^∗^ *p* value by linear mixed model.

**FIGURE 5:**
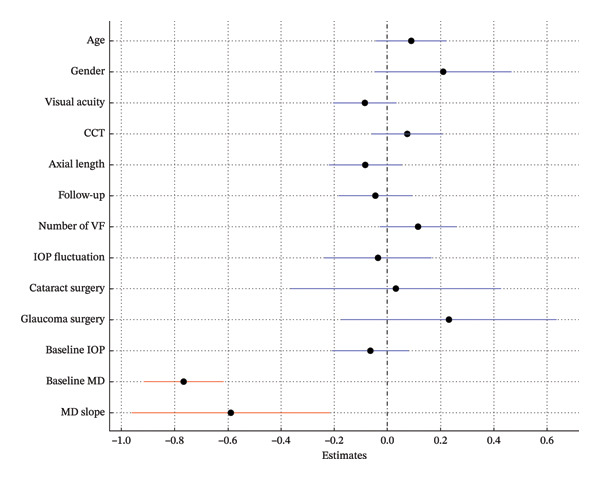
Forest plot showing Model 4, which includes baseline visual field mean deviation (MD) and baseline intraocular pressure (IOP). Dots indicate the estimates for the RMSE of the total deviation value (TDV); bars represent 95% confidence intervals. A lower baseline MD and steeper MD slope were significantly associated with increased VF variability. Red markers indicate statistically significant variables (*p* < 0.05). Abbreviations: CCT (central corneal thickness), IOP (intraocular pressure), RNFL (retinal nerve fiber layer), VF (visual field).

**FIGURE 6:**
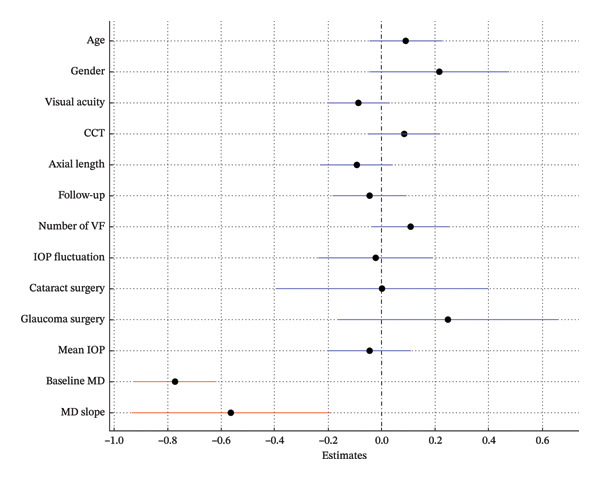
Forest plot showing Model 5, which includes baseline visual field mean deviation (MD) and mean intraocular pressure (IOP). Dots indicate the estimates for the RMSE of the total deviation value (TDV); bars represent 95% confidence intervals. A lower baseline MD and steeper MD slope were significantly associated with increased VF variability. Red markers indicate statistically significant variables (*p* < 0.05). Abbreviations: CCT (central corneal thickness), IOP (intraocular pressure), RNFL (retinal nerve fiber layer), VF (visual field).

**FIGURE 7:**
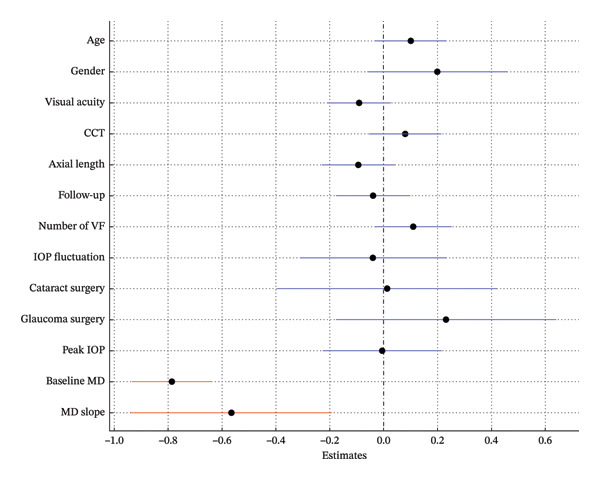
Forest plot showing Model 6, which includes baseline visual field mean deviation (MD) and peak intraocular pressure (IOP). Dots indicate the estimates for the RMSE of the total deviation value (TDV); bars represent 95% confidence intervals. A lower baseline MD and steeper MD slope were significantly associated with increased VF variability. Red markers indicate statistically significant variables (*p* < 0.05). Abbreviations: CCT (central corneal thickness), IOP (intraocular pressure), RNFL (retinal nerve fiber layer), VF (visual field).

In Model 4, which included baseline MD and baseline IOP, a lower baseline MD and a steeper MD slope were associated with increased long‐term VF variability (*p* < 0.001 and *p* = 0.003, respectively; Table 5; Figure 5). In Model 5, which included baseline MD and mean IOP, a lower baseline MD and a steeper MD slope were associated with increased long‐term VF variability (*p* < 0.001 and *p* = 0.004, respectively; Table 6; Figure 6). In Model 6, which included baseline MD and peak IOP, a lower baseline MD and a steeper MD slope were associated with increased long‐term VF variability (*p* < 0.001 and *p* = 0.004, respectively; Table 7; Figure 7).

**TABLE 5:** Relationship between long‐term pointwise variability and predictive factors of Model 4 (including baseline mean deviation and baseline intraocular pressure).

	Estimate	Standard error	*p* value^∗^
Age	0.090	0.068	0.190
Gender	0.208	0.131	0.118
Visual acuity	−0.085	0.06	0.157
Central cornea thickness	0.075	0.068	0.277
Axial length	−0.084	0.069	0.229
Length of follow‐up	−0.044	0.07	0.531
Number of visual field	0.115	0.073	0.120
Intraocular pressure fluctuation	−0.033	0.103	0.750
Cataract surgery during the follow‐up	0.031	0.201	0.877
Glaucoma surgery during the follow‐up	0.232	0.207	0.264
Baseline intraocular pressure	−0.064	0.074	0.384
Baseline mean deviation	−0.766	0.077	< 0.001
Mean deviation slope	−0.589	0.191	0.003

^∗^ *p* value by linear mixed model.

**TABLE 6:** Relationship between long‐term pointwise variability and predictive factors of Model 5 (including baseline mean deviation and mean intraocular pressure).

	Estimate	Standard error	*p* value^∗^
Age	0.091	0.069	0.189
Gender	0.216	0.134	0.113
Visual acuity	−0.085	0.06	0.159
Central cornea thickness	0.086	0.068	0.210
Axial length	−0.092	0.068	0.180
Length of follow‐up	−0.044	0.070	0.534
Number of visual field	0.109	0.072	0.135
Intraocular pressure fluctuation	−0.022	0.109	0.842
Cataract surgery during the follow‐up	0.002	0.203	0.994
Glaucoma surgery during the follow‐up	0.249	0.209	0.237
Mean intraocular pressure	−0.045	0.079	0.574
Baseline mean deviation	−0.771	0.079	< 0.001
Mean deviation slope	−0.562	0.190	0.004

^∗^ *p* value by linear mixed model.

**TABLE 7:** Relationship between long‐term pointwise variability and predictive factors of Model 6 (including baseline mean deviation and peak intraocular pressure).

	Estimate	Standard error	*p* value^∗^
Age	0.100	0.068	0.145
Gender	0.198	0.133	0.142
Visual acuity	−0.091	0.06	0.134
Central cornea thickness	0.081	0.068	0.237
Axial length	−0.094	0.069	0.177
Length of follow‐up	−0.038	0.07	0.586
Number of visual field	0.110	0.073	0.136
Intraocular pressure fluctuation	−0.040	0.138	0.776
Cataract surgery during the follow‐up	0.013	0.208	0.950
Glaucoma surgery during the follow‐up	0.232	0.209	0.271
Peak intraocular pressure	−0.005	0.112	0.964
Baseline mean deviation	−0.785	0.076	< 0.001
Mean deviation slope	−0.565	0.191	0.004

^∗^ *p* value by linear mixed model.

## 4. Discussion

The present study specifically investigated patients with NTG to elucidate the relationship among IOP parameters, structural characteristics, and functional variability. To our knowledge, no previous study has comprehensively analyzed the factors associated with long‐term variability in VF testing, specifically in patients with NTG, making this study the first.

Our group previously investigated long‐term VF variability in patients with primary open‐angle glaucoma (POAG) and demonstrated that greater IOP fluctuation, worse baseline VF damage, and faster functional deterioration were significantly associated with increased VF variability [12]. Although the present study shares a similar analytical framework with that prior work, it was intentionally designed to address an important limitation by restricting the study population to treatment‐naïve NTG patients with baseline IOP ≤ 21 mmHg. In contrast to the previous POAG cohort, which included patients with higher IOP levels and those receiving IOP‐lowering therapy, the current study excluded eyes exposed to elevated IOP or prior treatment, thereby isolating disease‐related functional instability inherent to NTG.

In recent years, OCT has arisen as an indispensable imaging modality in glaucoma care, providing objective, noninvasive, and highly reproducible measurements of the RNFLT [13–16]. Progressive RNFL thinning has further been well documented to correlate closely with functional decline, while insufficient structural reserve is thought to contribute to greater instability in VF measurements. Thus, integrating structural assessments using OCT with functional testing is considered the best practice for the modern management of glaucoma [14–16].

In the present study, we specifically investigated whether baseline RNFLT measured using OCT was associated with long‐term VF variability. Unlike the study by Rabiolo et al., our study incorporated the RNFLT as a key structural parameter in the evaluation of long‐term VF variability, enabling direct assessment of how structural damage influences functional instability in the NTG. Notably, a thinner baseline RNFLT was significantly associated with greater long‐term VF variability. This finding underscores the clinical importance of OCT evaluation in NTG; patients presenting with a thinner RNFL at baseline may warrant closer VF monitoring, as structural compromise may predispose them to more unstable functional measurements.

Along with the RNFLT, we also found that a lower baseline and mean IOP were associated with greater long‐term VF variability. Previous studies have shown that the long‐term variability in VF testing may be influenced not only by test‐related factors, such as false‐positive and false‐negative responses or test duration, but also by various patient‐related factors, including visual acuity, race, disease severity, learning effect, fatigue, media opacity, IOP fluctuation, baseline MD, history of glaucoma surgery, cognitive decline, and length of follow‐up [5–8, 17, 18]. Gardiner et al. further reported that as glaucoma progresses and threshold values at each VF point decrease, the variability in VF testing results increases [19].

Consistent with previous reports, including the work of Rabiolo et al. [6], the present study confirmed the associations between greater IOP fluctuations, worse baseline MD, and a steeper MD slope with increased long‐term VF variability in our NTG cohort. However, the significant association between lower baseline and mean IOP and VF variability observed in the present study was notably absent in the POAG population reported by Rabiolo et al. [6]. This difference likely reflects the distinctive pathophysiology of NTG, which is characterized by lower IOP and unique structural vulnerabilities.

In general glaucoma populations, lower IOP is considered protective against disease worsening; however, because this study exclusively evaluated NTG, the observed association of greater VF variability with lower baseline and mean IOP is an important finding. These results highlight that in NTG, factors beyond the absolute IOP level—particularly the intrinsic structural vulnerability of the optic nerve head—may exert a more dominant influence on functional instability than pressure magnitude itself.

Notably, eyes with NTG have been reported to exhibit a thinner lamina cribrosa (LC) than eyes with POAG at similar disease stages, reflecting reduced structural resilience at the optic nerve head [20, 21]. Such structural fragility likely amplifies the adverse effects of small IOP variations, leading to greater long‐term VF measurement instability [22, 23]. In NTG, a compromised LC may deform under transient pressure changes, thereby impairing axonal transport and circulation and promoting disease progression with greater VF variability [20, 23]. The combination of lower baseline/mean IOP and LC vulnerability in the NTG provides a plausible mechanistic explanation for the significant association between IOP fluctuations and VF variability identified in the present study. Clinically, this finding emphasizes the need for more frequent and individualized VF monitoring in patients with NTG to promptly detect subtle progression.

In their study, Rabiolo et al. adopted pointwise approaches and used an exponential regression of the threshold sensitivities at each test location to the estimate pointwise RMSE [6]. Additional models, including linear and logistic regressions, were further used to validate the findings [6]. In contrast, our study exclusively employed a pointwise approach using TDVs rather than threshold sensitivities. At each VF location, the TDVs regressed linearly over time, and the RMSE between the observed and predicted values was calculated to represent the long‐term VF variability. Unlike previous studies, our analysis incorporated clinical parameters such as RNFLT, IOP metrics, and VF progression rate, into a multivariate framework, making the results more applicable to real‐world clinical management.

Rabiolo et al. further reported that faster rates of glaucoma progression, measured using a pointwise exponential regression model known as the Glaucoma Rate Index (GRI), were associated with greater VF fluctuation [6]. In the present study, a similar trend was observed: steeper MD slopes, reflecting more rapid functional deterioration, were significantly correlated with increased long‐term VF variability. Although different methods have been used to quantify progression (GRI versus linear MD slope), both findings indicate that eyes undergoing faster glaucomatous damage may exhibit greater instability in VF measurements. This may be attributable to the fact that such eyes progress from early to more advanced stages, where VF fluctuation tends to increase.

Overall, this study included patients with early to moderate stage of NTG, as reflected by the baseline MD observed in the study population (mean baseline MD: −4.49 ± 4.10 dB/year). Consequently, these data may not fully capture the extent or pattern of long‐term VF variability in patients with advanced glaucoma. Future studies comparing VF variability between early and advanced stages of glaucoma should thus be conducted to provide further insight into how disease severity influences fluctuation in functional testing.

Overall, this study employed a linear regression model to analyze the changes in VF loss over time. In pointwise trend‐based analysis, various regression models, including exponential, logistic, and polynomial models, can be applied to evaluate the progression at each VF location. Prior studies have further suggested that nonlinear models may better capture disease progression, particularly in patients with advanced glaucoma approaching perimetric blindness [24]. Given this, future studies incorporating alternative regression models, such as exponential or logistic approaches, are warranted to validate and enhance the generalizability of our findings.

Although our discussion highlights the potential role of LC structural vulnerability in increasing VF variability in the NTG, LC parameters were not directly measured in the present study. Future investigations incorporating direct LC morphological assessments may help clarify their contribution to functional variability and further elucidate the underlying pathophysiology of NTG.

In conclusion, this study demonstrated that long‐term VF variability in patients with NTG was significantly associated with lower baseline RNFLT, lower baseline and mean IOP, greater IOP fluctuation, worse baseline VF damage, and faster VF progression. These results further underscore the importance of interpreting changes in VF in the combined context of structural integrity and IOP‐related parameters. By explicitly distinguishing NTG from POAG and focusing on treatment‐naïve eyes with low IOP, our findings emphasize that VF variability in NTG reflects intrinsic structural susceptibility rather than pressure elevation alone. Incorporating OCT‐derived RNFLT measurements and comprehensive IOP profiling into clinical decision‐making may facilitate individualized management and timely therapeutic adjustments, particularly in patients with low IOP and unstable disease courses. Patients with NTG presenting with thinner baseline RNFLT or rapid functional decline may therefore require more frequent VF examinations to distinguish true progression from fluctuation, even when IOP appears well controlled.

## Funding

This work was supported by a 2 year Research Grant of Pusan National University.

## Conflicts of Interest

The authors declare no conflicts of interest.

## Data Availability

The data that support the findings of this study are available on request from the corresponding author. The data are not publicly available due to privacy or ethical restrictions.
